# Bibliometric analysis of research hotspots and trends on the relationship between the gut microbiota and depression from 2020 to 2024

**DOI:** 10.3389/fmicb.2024.1479703

**Published:** 2024-11-11

**Authors:** Dingwen Xu, Jijun Wu, Zhihua Lu, Xu Zhao, Yang Feng, Weicai Zhang, Shenglu Jiang, Lingling Zhang, Ting Wang, Zhenxiong Zhao

**Affiliations:** ^1^Department of Clinic, School of Medicine, Yangzhou Polytechnic College, Yangzhou, China; ^2^Department of Interventional Radiology, Zhongshan Torch Development Zone People's Hospital, Zhongshan, China; ^3^Department of Geriatrics, The Third Hospital of Santai, Santai, China; ^4^Taizhou Central Hospital (Taizhou University Hospital), Taizhou, China; ^5^School of Medicine, Taizhou University, Taizhou, Zhejiang, China

**Keywords:** bibliometric analysis, gut microbiota, depression, publication, authors

## Abstract

**Introduction:**

In recent years, an increasing body of research has illustrated a strong correlation between gut microbiota and depression. However, there has yet to be a comprehensive discussion or summary of the latest advancements and trends in this field.

**Methods:**

We retrieved research articles focused on gut microbiota and depression through the WOS database from 2020 to 2024, using visual text analysis tools such as CiteSpace and VOSviewer.

**Results:**

The literature on the relationship between gut microbiota and depression surged from 396 papers in 2020 to 711 by 2024. During this period, the journal with the highest publication rate was Nutrients. China led the countries in contributions, while University College Cork topped the institutions. Kenji Hashimoto emerged as the most prolific author. The most cited paper was authored by Cryan JF et al., published in 2019 in Physiol Rev. The keywords “gut microbiota,” “depression,” and “anxiety” appeared most frequently, while recent years saw explosive increases in terms such as “growth performance,” “receptors,” “depression-like phenotypes,” “stress response,” “gastrointestinal symptoms,” “reliability,” and “neurogenesis.”

**Discussion:**

Our article displayed the overview of the relationship between the gut microbiome and depression from 2020 to 2024 using bibliometric methods, providing perspectives and research hotspots for studies exploring the correlation between the gut microbiome and depression.

## Introduction

Depression is a chronic and recurrent mental disease characterized by symptoms such as poor mood, loss of interest, sleep and eating disorders, and decreased volitional activity. In severe cases, there may be hallucinations, delusions, and other symptoms, and in extreme cases, there may be suicidal thoughts or behaviors (Monroe and Harkness, 2022). Depression is projected to become the leading cause of illness globally by 2030. According to the World Health Organization (WHO), approximately 300 million people worldwide suffer from depression (Yuan et al., 2024). Adolescents are in a critical period of rapid social, emotional, and cognitive development and life transformation, and the incidence of adolescent depression has increased dramatically in recent years. Over a lifetime, depression is about twice as common in women as in men (Bore et al., 2024). Postpartum depression affects 7–30% of women worldwide and approximately 45% of women in some poor areas (Prifti et al., 2024). In addition, the global spread of novel coronavirus (COVID-19) in recent years has disrupted people’s normal life and work order, resulting in a significant increase in the incidence and recurrence rate of depression. Therefore, research on depression since COVID-19 is necessary (Herrmann et al., 2024).

The etiology of depression is not completely clear, but clinical studies have shown that the pathogenesis of depression is related to a variety of factors, such as biological factors, including genetics depression-related genes, neurobiochemistry, and regeneration (Lin et al., 2024). According to the gut microbiota hypothesis, abnormalities of the gut microbiota can directly induce depression. Gut microbiota is a group of microorganisms inhabiting in the human digestive tract, which is regarded as a “dynamic organ” and plays an important role in brain function and emotional regulation (Ashique et al., 2024). Under normal circumstances, these bacteria remain relatively stable, and they work together to maintain the integrity of the intestinal mucosal barrier and the balance of the immune system, prevent harmful substances from entering the blood circulation through the intestinal wall, prevent excessive inflammation, and maintain the health of the body. When the gut microbiota is dysregulated, it may trigger disorders in brain function and related emotional regulation (Xu et al., 2024). On the one hand, depressive-like behavior can be induced by transplanting gut microbiota from depressed patients into rats lacking gut microbiota (Cheung et al., 2019). On the other hand, inducing depression-like behavior in rodents led to a decrease in the abundance of their gut microbiota (Winter et al., 2018). Antidepressants are the treatment of choice for patients with moderate-to-severe depression. Due to individual differences, only 5–20% of patients who take antidepressants will achieve complete and lasting remission, and approximately 30% of patients do not achieve the expected treatment effect (Vas et al., 2023). Moreover, long-term use of antidepressants may cause side effects such as weight gain, heavy sweating, and sexual dysfunction. More than 50% of these patients will experience withdrawal reactions when they stop taking antidepressants, which may further aggravate the degree of depression (Gill et al., 2020). Therefore, the safety and personalized measures of intestinal microbiota-based adjuvant treatment of depression, such as dietary intervention, microecological agents, and fecal transplantation, deserve attention.

With a deepening understanding of the relationship between depression and gut microbiota, understanding the hot topics at home and abroad is helpful to grasp the frontier trend in this field. Bibliometric analysis employs quantitative research methods such as mathematics and statistics to depict, evaluate, and predict the structure, characteristics, and patterns of literature studies. It assesses the interconnections and impacts of published articles and their citations to grasp research directions effectively. This study uses the bibliometric method to analyze the literature published from 2020 to 2024 on the correlation between gut microbiota and depression, so as to provide a reference for the study of the pathogenesis of depression and the prevention and treatment of depressions.

## Methods

### Data source and search strategy

**Figure 1:**
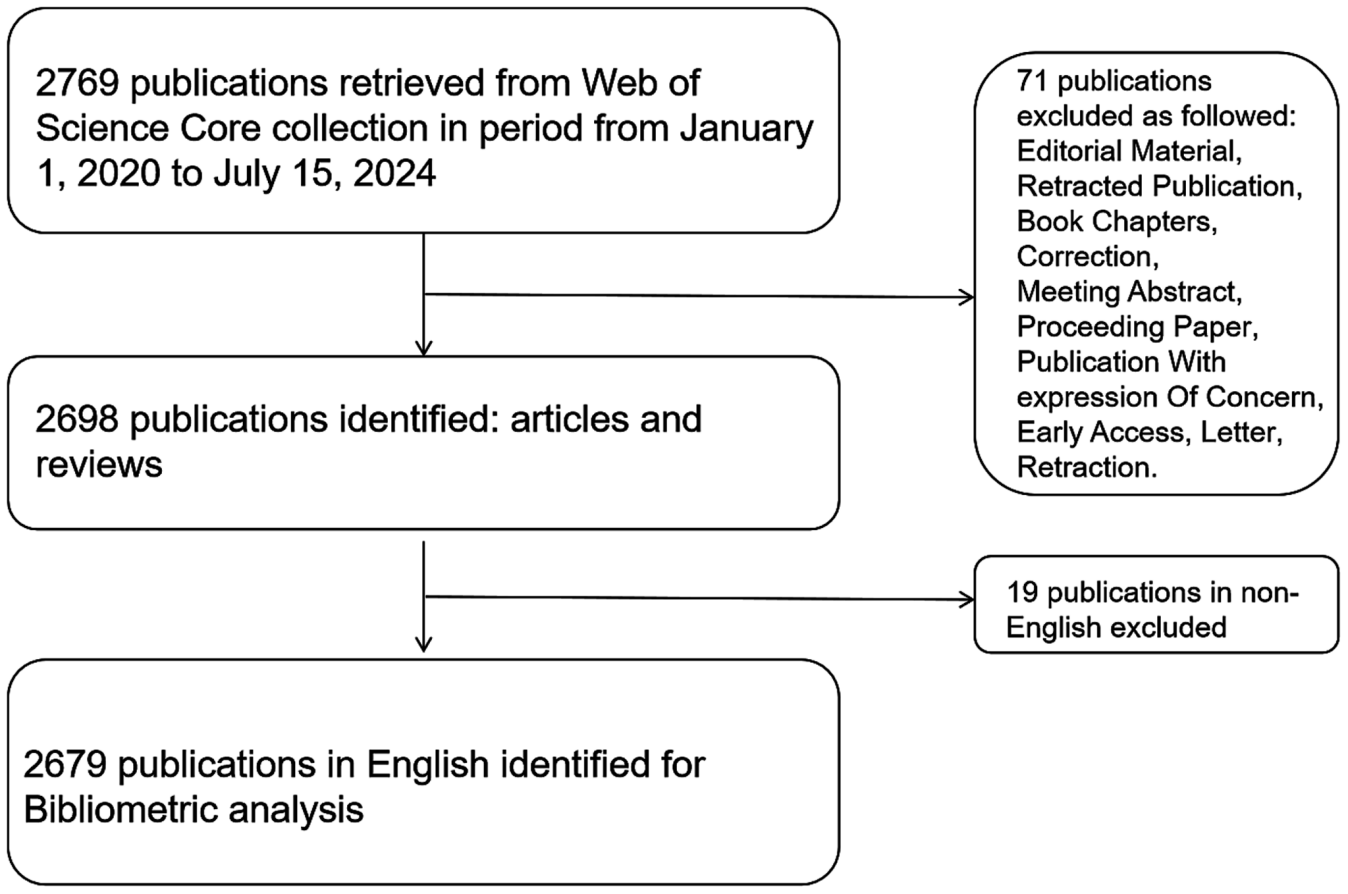
Analysis flowchart of gut microbiota and depression.

Subject term searches were performed in the Web of Science core collection. Retrieval time is from 1 January 2020 to 15 July 2024. The language is English. The search strategy was as follows: TS = (gut microflora OR gut microorganism OR intestinal microflora OR intestinal microorganism OR microflora OR probiotics OR synbiotics OR prebiotics OR gut microb* OR intestinal microb*) AND TS = (depression OR depressions OR depressed OR despondent OR depressive OR gloomy). Inclusion criteria were as follows: (1) the type of literature was original articles and reviews. Exclusion criteria were as follows: (1) The document language is not English and (2) Editorial Material, Retracted Publication, Book Chapters, Correction, Meeting Abstract, Proceeding Paper, Early Access, Letter, Retraction, and Publication With Expression Of Concern. Two researchers read the abstracts or full texts and screened them one by one. Disagreements were resolved by discussion or consultation with a third researcher (see Figure 1).

### Data analysis and visualization

Retrieve publication dates, citation counts, author details, institutional affiliations, countries, keywords, and other pertinent information from the Web of Science Core Collection database. Using VOSviewer 1.6.18 software, we extracted the literature, research institutions, national, and keywords. We then generate collaboration networks for countries, research institutions, and authors, along with high-frequency keyword and clustering network visualizations. Nodes represent the analyzed elements such as countries, institutions, and journals, and different colors indicate different clusters. Node size can reflect the frequency of element occurrence, and the connection between nodes represents cooperation and co-occurrence. Scimago Graphica 1.0.43 was used to display the national cooperation network on the map. The CiteSpace 5.7 R5 software was used for co-cited reference analysis, keyword analysis, and burst analysis.

## Results

### Analysis of annual publications

**Figure 2:**
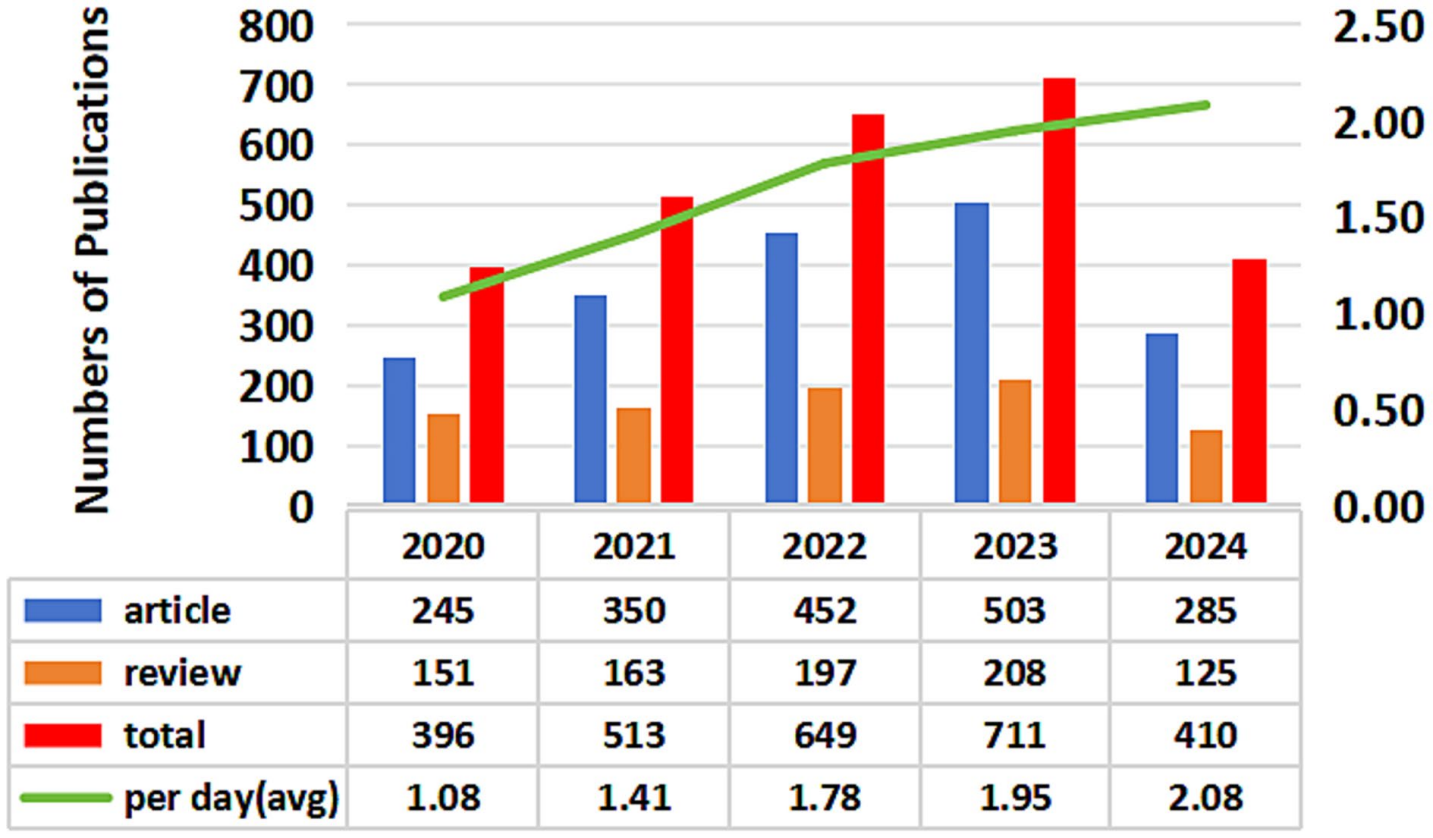
Publications of gut microbiota and depression indexed from 2020 to 2024.

According to our search strategy, the bibliometric analysis included 2,679 publications regarding gut microbiota and depression. This comprised 1,835 articles (68.5%) and 844 reviews (31.5%). The annual publication numbers from 2020 to 2024 concerning gut microbiota and depression are illustrated in Figure 2. Overall, the publication count has increased each year, rising from 396 articles in 2020 to 711 articles in 2022. In addition, by 15 July 2024, a total of 285 articles have been published. Calculating the average daily publications, the rate rose from 1.08 articles per day in 2020 to 2.08 articles per day in 2024. This analysis indicates a rapid growth in research interest regarding gut microbiota and depression over the past 5 years, suggesting that this trend may continue in the future.

### Analysis of journal

**Figure 3:**
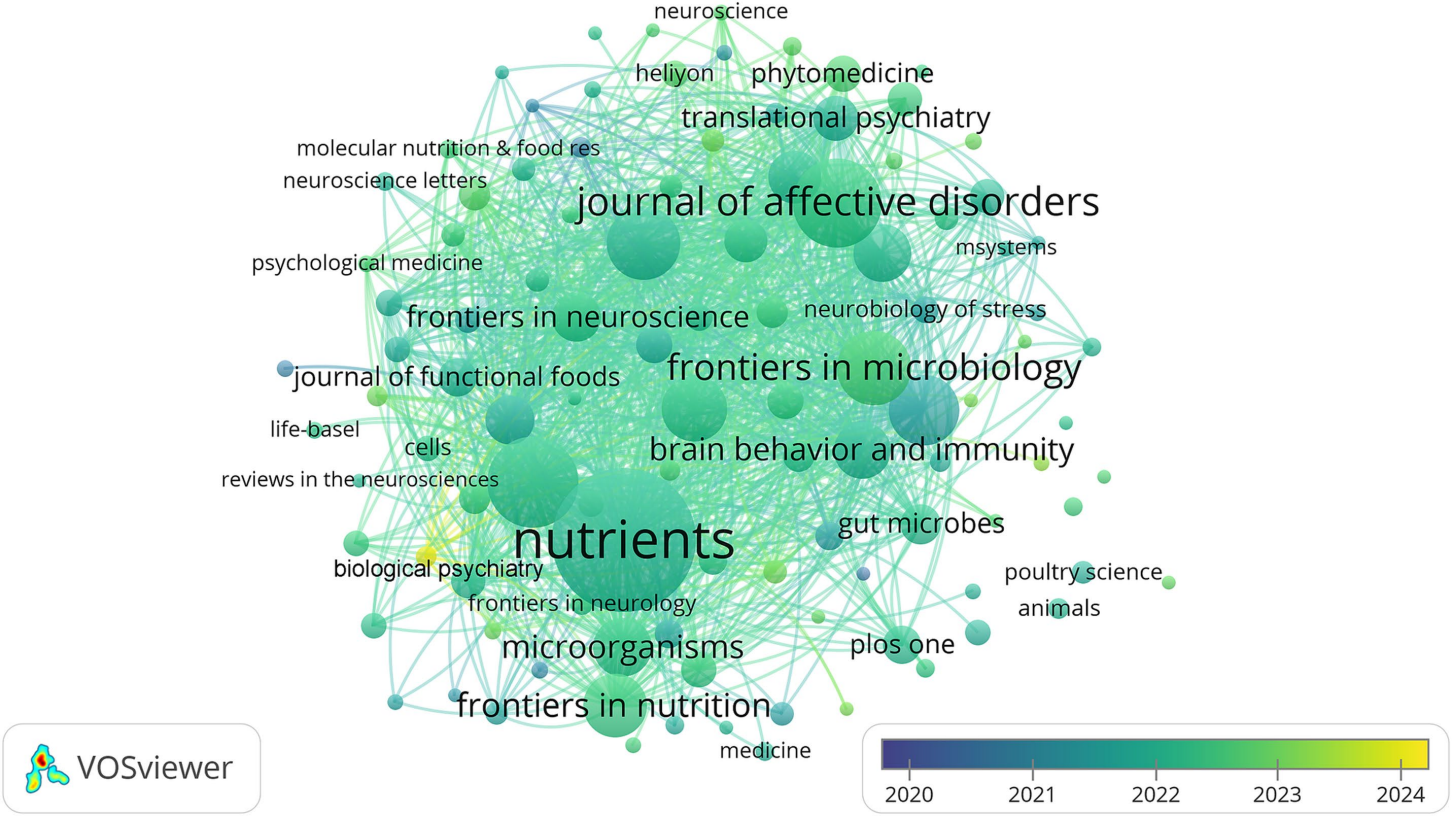
Analysis of journal from 2020 to 2024.

A total of 778 journals published 2,679 studies on gut microbiota and depression. The top 10 journals published 598 articles, accounting for 22.3% of all publications (see Table 1). Among them, “Nutrients” led with 136 articles, followed by the “International Journal of Molecular Sciences” (71 articles), “Journal of Affective Disorders” (69 articles), “Frontiers in Microbiology” (54 articles), and “Frontiers in Psychiatry” (53 articles), with “Scientific Reports” contributing 50 articles. All these prolific journals boast impact factors exceeding 3.0, with the majority surpassing 5.0. Furthermore, visualization overlap analysis using VOSviewer software on journals with at least five published articles (*n* = 106) revealed that “Biological Psychiatry” has emerged as a significant journal related to gut microbiota and depression in recent years, publishing nine articles with an average publication year of 2023.78 (see Figure 3).

**Table 1:** Top 10 productive journals of gut microbiota and depression.

Journals	Documents	Average impact factor (in the past 5 years)
Nutrients	136	5.8
International Journal of Molecular Sciences	71	5.6
Journal of Affective Disorders	69	5.4
Frontiers in Microbiology	54	5.1
Frontiers in Psychiatry	53	3.9
Scientific Reports	50	4.3
Food and Function	45	5.6
Frontiers in Nutrition	43	4.4
Microorganisms	39	4.5
Frontiers in Cellular and Infection Microbiology	38	5.1

### Analysis of country and organization

**Figure 4:**
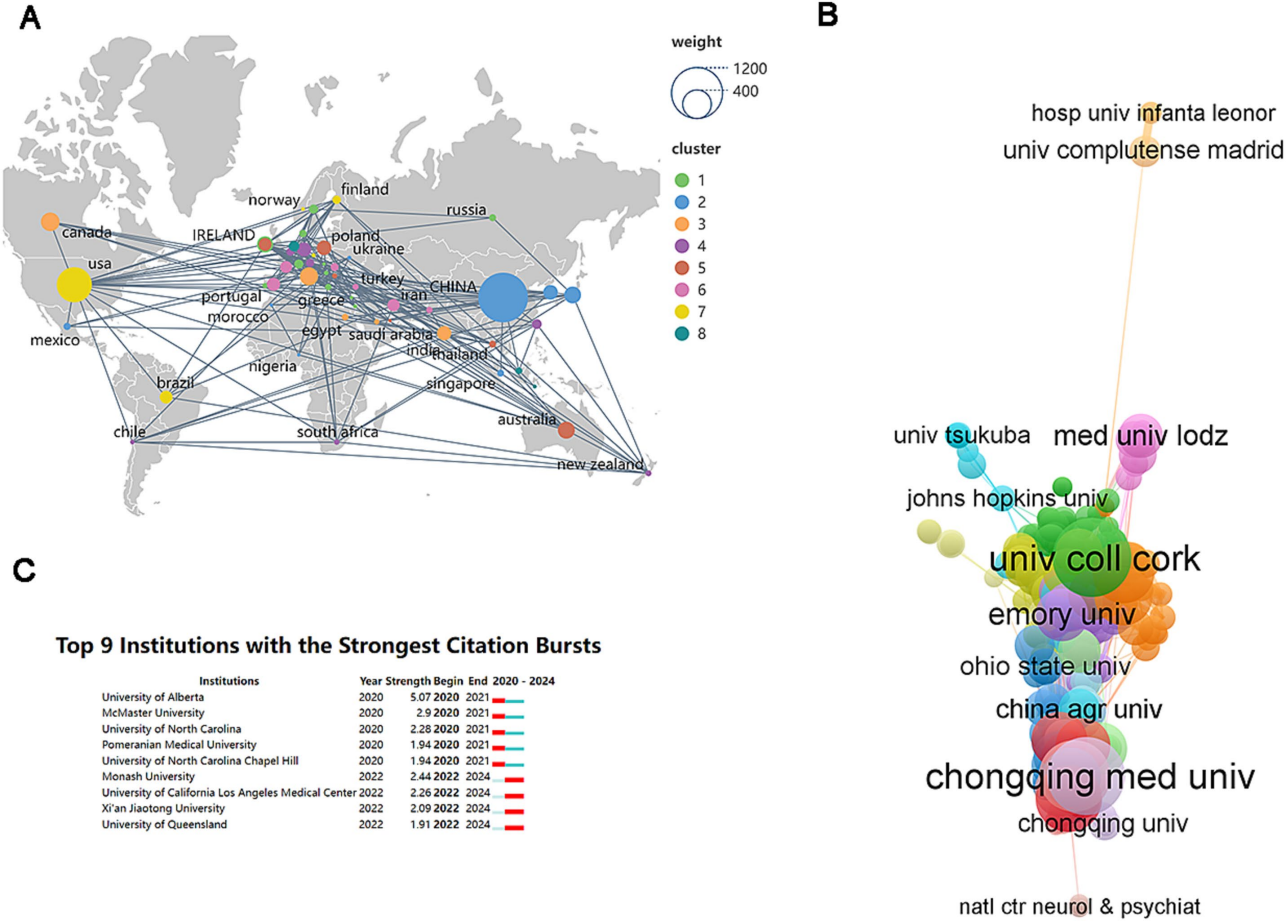
Analysis of country and organization. **(A)** The publications density map of country. **(B)** The collaboration network of institutions. **(C)** Top nine institutions with the strongest citation bursts.

A total of 2,679 papers originated from 98 countries/regions. The top 10 countries accounted for 92.8% of all 2,679 papers. The selection criteria for 55 countries/regions required a minimum publication count of “5 papers.” Figure 4A clearly shows that the United States and China play significant roles among all countries/regions involved in this field. Research from 3,422 institutions contributed to the 2,679 papers. There were 317 institutions with at least five published papers, and the collaboration network of these institutions was constructed using VOSviewer (see Figure 4B). The top 10 institutions by publication count are listed in Table 2. Notably, the six most productive institutions are located in China, followed by Australia (with two institutions). It is worth mentioning that while the UK’s research institution, University College Cork, is the only one to rank in the top 10, it leads in both paper publication and citation counts. Although the United States ranks second globally for paper publications, it does not have institutions among the top 10. This indicates that research on gut microbiome and depression in the United States is relatively dispersed and balanced. Figure 4C displays the institutions with the most significant increase in publication numbers. Monash University, the University of California Los Angeles Medical Center, and the University of Queensland have published the highest number of papers in this field over the past 2 years.

**Table 2:** Top 10 institutions by publication count.

Organization	Country	Documents	Citations
University College Cork	Britain	60	2,612
Chongqing Medical University	China	59	1,279
Zhejiang University	China	50	942
Chiba University	Japan	43	1,372
Deakin University	Australia	39	1,438
Chinese Academy of Sciences	China	35	911
University of Melbourne	Australia	34	1,812
Shanghai Jiao Tong University	China	32	300
Capital Medical University	China	31	585
Southern Medical University	China	30	318

### Analysis of authors

**Figure 5:**
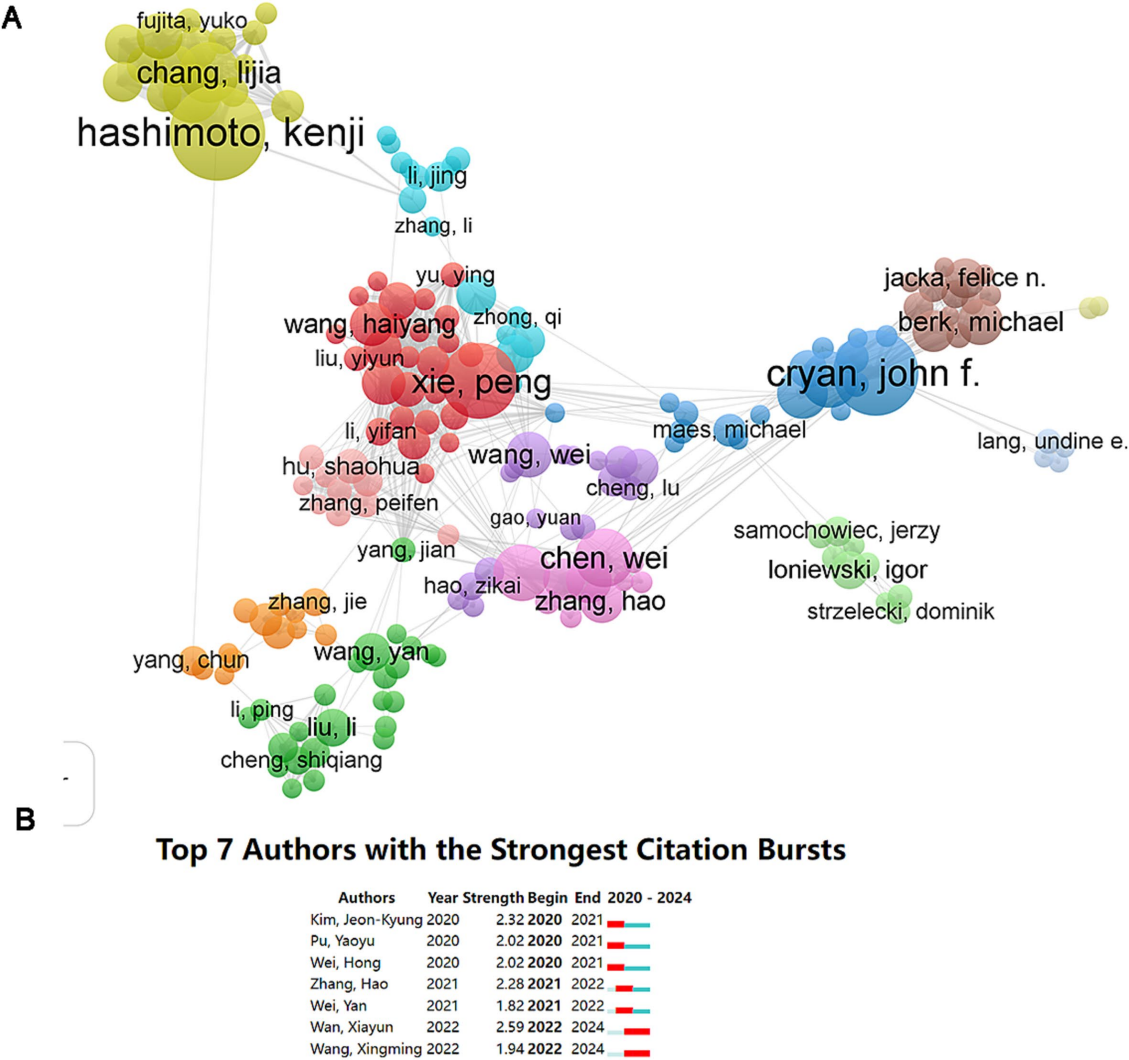
Analysis of authors. **(A)** The collaboration network of authors. **(B)** Top seven authors with the strongest citation bursts.

Among 14,539 authors, 235 have published at least five papers on gut microbiota and depression. Among the top 10 authors with the highest publication counts, three are from China, three from Japan, three from the United Kingdom, and one from South Korea (see Table 3). Based on the number of papers, the author with the most publications is Kenji Hashimoto from Chiba University (*n* = 43), followed by John F. Cryan from University College Cork (*n* = 37) and Peng Xie from Chongqing Medical University (*n* = 31). John F. Cryan’s papers also received the highest total citations (1,725 times). A collaboration network analysis of authors with at least five publications was presented using VOSviewer software (see Figure 5A). Authors belonging to the same cluster/color, such as Kenji Hashimoto and Lijia Chang, exhibit strong collaborative relationships. However, interaction between different clusters/colors is notably limited, indicating restricted collaboration among various research teams. Moreover, Figure 5B illustrates the rapidly increasing number of publications in this field. Wan Xiayun and Wang Xingming are the authors with the fastest growth in published papers over the past 2 years.

**Table 3:** Top 10 authors with the highest publication counts.

Authors	Publications	Citations	Organization	Country
Hashimoto, Kenji	43	1,372	Chiba University	Japan
Cryan, John F.	37	1,725	University College Cork	Britain
Xie, Peng	31	695	Chongqing Medical University	China
Chang, Lijia	23	1,068	Chiba University	Japan
Qu, Youge	22	742	Chiba University	Japan
Chen, Wei	22	573	Guizhou Normal University	China
Wang, Gang	21	594	Capital Medical University	China
Dinan, Timothy G.	21	1,246	University College Cork	Britain
Clarke, Gerard	18	771	University College Cork	Britain
Kim, Dong-Hyun	17	335	Kyung Hee University	Korea

### Analysis of co-cited references

**Figure 6:**
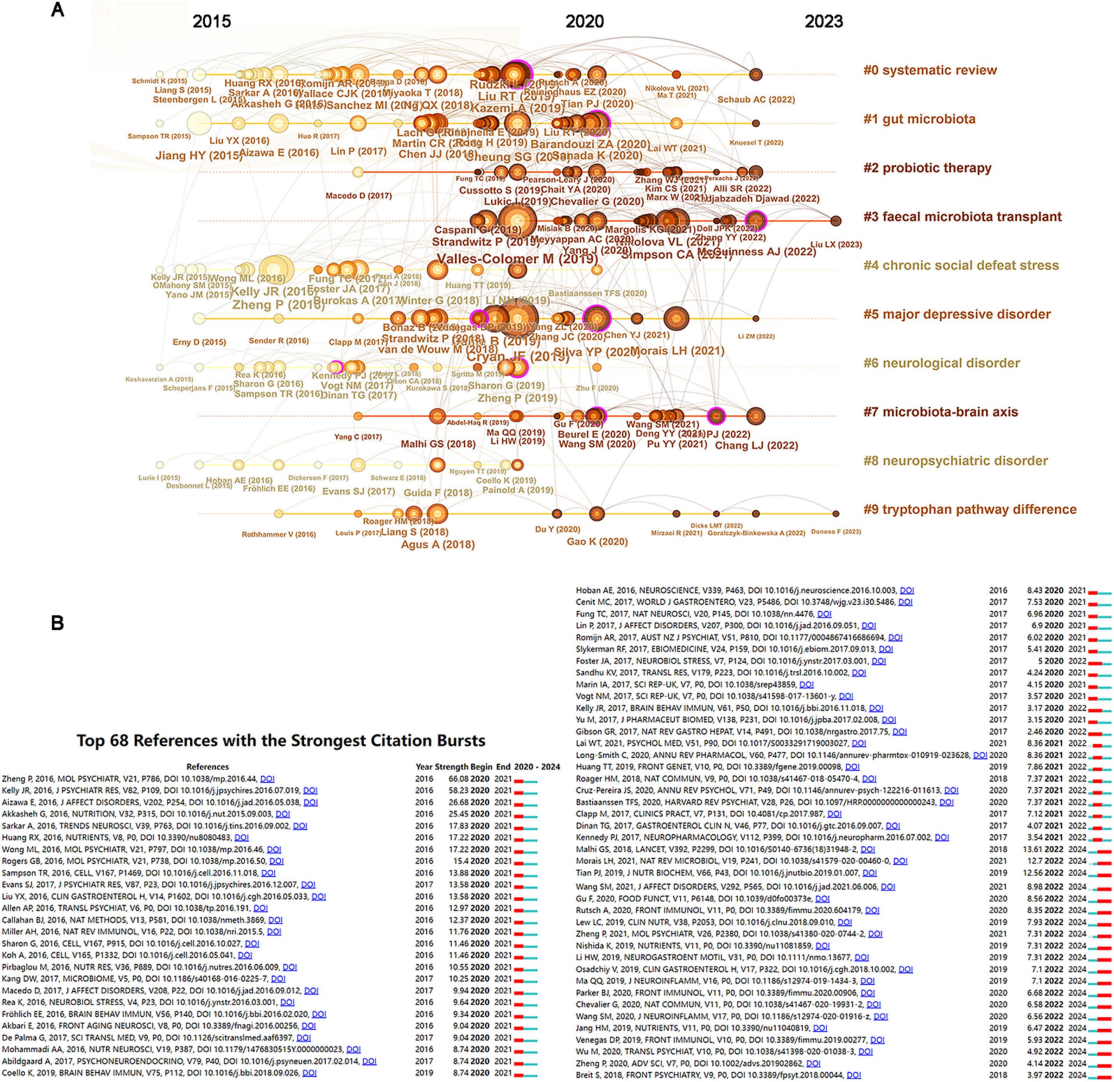
Analysis of co-cited references. **(A)** The timeline view of co-cited references. **(B)** Top 68 references with the strongest citation bursts.

Table 4 lists the top 10 references with the highest citation frequencies in studies on gut microbiota and depression. The most cited article is by Cryan et al. (2019), published in 2019 in Physiol Rev, with 400 citations. Following this, the article by Valles-Colomer et al. (2019), published in 2019 in Nat Microbiol, received 362 citations, while Zheng et al. (2016) published a paper in Mol Psychiatry in 2016, which garnered 215 citations. A co-citation visualization analysis of references conducted using CiteSpace clustered co-cited references into categories, including systematic review, gut microbiota, probiotic therapy, fecal microbiota transplant, chronic social defeat stress, major depressive disorder, neurological disorder, microbiota–brain axis, neuropsychiatric disorder, and tryptophan pathway difference, totaling 10 clusters. A timeline view displayed the evolution of co-cited references over time (Figure 6A). In the timeline view, nodes of different colors in the same row represent different years; left-side nodes indicate earlier keywords, while right-side nodes indicate more recent keywords. Lines on the same horizontal level represent the collective citations of all clusters in that row. As shown in Figure 6A, chronic social defeat stress represents an early research direction in the field, while probiotic therapy and fecal microbiota transplant are currently popular research areas. Figure 6B indicates that the most highly cited publication since 2020, authored by Zheng et al. (2016), was published in 2016 in “Mol Psychiatry.” Following closely is the paper by Kelly et al. (2016), published in 2016 in “J PSYCHIATR RES.”

**Table 4:** Top 10 references with the highest citation frequencies.

Title	Journal	First author	Year	Total citation frequency
The microbiota–gut–brain axis	Physiol Rev	Cryan JF	2019	400
The neuroactive potential of the human gut microbiota in quality of life and depression	Nat Microbiol	Valles-Colomer M	2019	362
Gut microbiome remodeling induces depressive-like behaviors through a pathway mediated by the host’s metabolism	Mol Psychiatry	Zheng P	2016	215
The role of short-chain fatty acids in microbiota–gut–brain communication	Nat Rev Gastro Hepat	Dalile B	2019	196
Transferring the blues: depression-associated gut microbiota induces neurobehavioral changes in the rat	J Psychiatr Res	Kelly JR	2016	190
Systematic review of gut microbiota and major depression	Front Psychiatry	Cheung SG	2019	184
Effect of probiotic and prebiotic vs. placebo on psychological outcomes in patients with major depressive disorder: a randomized clinical trial	Clin Nutr	Kazemi A	2019	175
The gut microbiota in anxiety and depression: a systematic review	Clin Psychol Rev	Simpson CA	2021	174
Prebiotics and probiotics for depression and anxiety: a systematic review and meta-analysis of controlled clinical trials	Neurosci Biobehav Rl	Liu RT	2019	168
Probiotic *Lactobacillus plantarum* 299v decreases kynurenine concentration and improves cognitive functions in patients with major depression: a double-blind, randomized, placebo-controlled study	Psychoneuroendocrino	Rudzki L	2019	160

### Analysis of keywords

**Figure 7:**
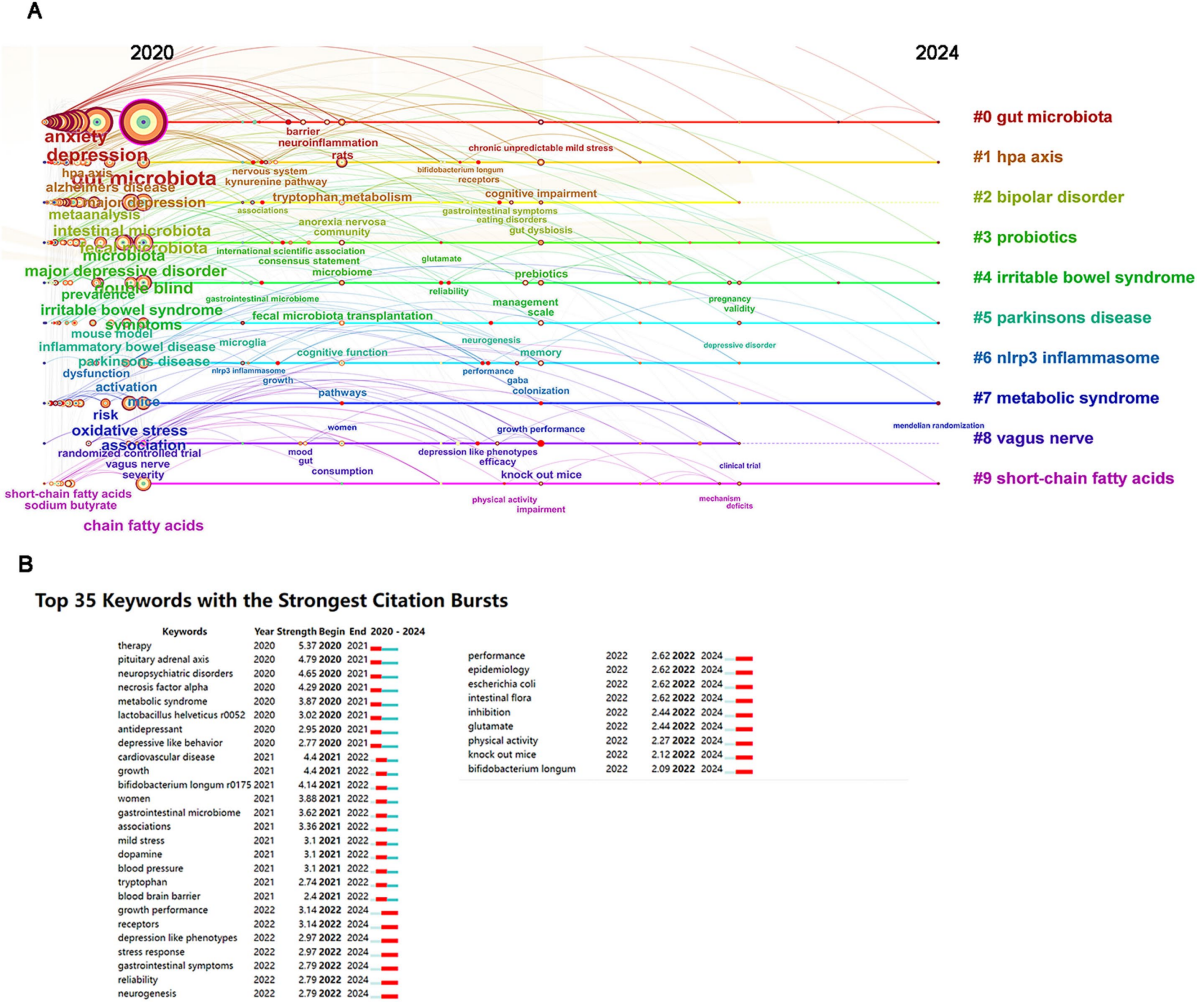
Analysis of keywords. **(A)** Keyword co-occurrence analysis. **(B)** Top 35 keywords with the strongest citation bursts.

The themes of interest in academia and the cutting-edge issues within specific fields reflect the latest research challenges and trends. We utilized CiteSpace to analyze keyword co-occurrence and clustering, aiming to capture the forefront of research in this domain. The keywords were classified into 10 categories: gut microbiota, Hypothalamic-pituitary-adrenal (HPA) axis, bipolar disorder, probiotics, irritable bowel syndrome, Parkinson’s disease, NLRP3 inflammasome, metabolic syndrome, vagus nerve, and short-chain fatty acids (see Figure 7A). The temporal evolution of keyword clustering indicates that HPA axis, irritable bowel syndrome, and metabolic syndrome are current research hotspots. We identified the top 20 keywords frequently appearing in the fields of gut microbiota and depression, with “gut microbiota” being the most prevalent (1,239 occurrences), followed by “depression” (664 occurrences) and “anxiety” (416 occurrences) (see Table 5). Figure 7B displays the 35 keywords with the strongest citation bursts. “Therapy” ranks as the strongest burst keyword in the field from 2020 to 2024 (strength: 5.37), followed by “pituitary adrenal axis” (strength: 4.79) and “neuropsychiatric disorders” (strength: 4.65). The recent 2 years highlight keyword bursts such as “growth performance,” “receptors,” “depression-like phenotypes,” “stress response,” “gastrointestinal symptoms,” “reliability,” and “neurogenesis.” These trends reflect the latest research developments.

**Table 5:** Top 20 keywords with the highest frequencies.

Rank	Keywords	Frequency	Rank	Keywords	Frequency
1	gut microbiota	1,239	11	intestinal microbiota	242
2	depression	664	12	behavior	238
3	anxiety	416	13	health	217
4	brain	353	14	major depressive disorder	204
5	stress	319	15	microbiota	199
6	fecal microbiota	275	16	symptoms	190
7	gut–brain axis	273	17	association	177
8	inflammation	271	18	chain fatty acids	167
9	gut microbiome	259	19	irritable bowel syndrome	167
10	double blind	248	20	expression	157

## Discussion

The study of depression continues to reveal its devastating impact on global health. Characterized by enduring low mood and significant functional impairment, it calls for urgent attention (Monroe and Harkness, 2022). Recent estimates indicate that approximately 300 million individuals worldwide suffer from this illness. Alarmingly, the incidence of depression among adolescents has surged in recent years, necessitating comprehensive research into its underlying causes (Yuan et al., 2024). Existing literature highlights multifactorial etiology, with biological factors playing a crucial role. Genetic predispositions and neurobiochemical imbalances contribute significantly to the condition (Lin et al., 2024). Notably, the gut–brain axis has emerged as a pivotal area of focus, with research suggesting that gut microbiota abnormalities can provoke depressive symptoms (Ashique et al., 2024). This intricate relationship dictates the importance of maintaining microbiota balance for optimal mental health. In our research, analysis of annual publications result showed over this 5-year span, and the total number of published works has exhibited a consistent upward trend. Specifically, the total count of published articles escalated from 396 in 2020 to an impressive 711 articles by 2022. This quantifiable surge underscores a burgeoning research interest in the complex interplay between gut microbiota and mental health ailments, specifically depression. Given this trajectory, it stands to reason that this increasing focus on the gut–brain axis may persist, propelling further inquiries and investigations in the years to come.

Bibliometric analyses of recent literature from 2020 to 2024 reveal that notable journals, such as “Nutrients” and “Biological Psychiatry,” are at the forefront in contributing impactful studies, while China ranks prominently in terms of research output, indicating a robust research environment. In contrast, Australia is represented by two notable institutions, showcasing its involvement in this significant field of inquiry. The United States holds a commendable second position globally regarding the volume of papers produced; it surprisingly lacks representation in the top 10 institutional ranking. This observation suggests a dispersion of research efforts across various institutions in the United States focused on the intersection of gut microbiome and depression rather than concentration at a few leading entities.

The extensive author collaboration network highlights significant contributions across various institutions. The visual representation demonstrates that authors grouped within the same cluster, such as Kenji Hashimoto and Lijia Chang, showcase robust collaborative relationships. Conversely, interactions between disparate clusters reveal a marked limitation, indicative of reduced collaborative endeavors among varying research teams. Wan Xiayun and Wang Xingming emerge as authors exhibiting the most rapid increase in published works over the preceding 2 years, suggesting a burgeoning focus on this critical area of study. As novel methodologies and advancements in technology continue to evolve, it is likely that these authors will lead the forefront of innovation. The emerging areas of focus, particularly in connection with interdisciplinary approaches, might catalyze further investigations on the correlation between gut microbiota and depression.

The comprehensive analysis of co-cited references reveals significant trends in the academic landscape surrounding the intricate relationship between gut microbiota and depression. The seminal work by Cryan et al. (2019), published in the esteemed journal Physiol Rev in 2019, has amassed an impressive total of 400 citations, underscoring its foundational impact on subsequent research. Following closely is the influential paper authored by Valles-Colomer et al. (2019), also published in 2019 in Nature Microbiology, which achieved 362 citations. This highlights a burgeoning interest in the microbiome’s role in mental health. In addition, an earlier but significant contribution by Zheng et al. (2016), published in Molecular Psychiatry in 2016, has garnered 215 citations. These references exemplify key contributions to the ongoing discourse on gut–brain interactions. A co-citation visualization analysis reflects the multidisciplinary nature of the research landscape. A timeline view illustrates chronic social defeat stress emerges as a pioneering research focus, while currently, probiotic therapies and fecal microbiota transplants dominate the research agenda.

Utilizing the analytical tool CiteSpace, we conducted a thorough investigation into the co-occurrence and clustering of keywords, with the intention to delineate the cutting-edge developments within this specific domain of study. The trends noted in the last 2 years reveal an array of additional keyword bursts that warrant attention, including “growth performance,” “receptors,” “depression-like phenotypes,” “stress response,” “gastrointestinal symptoms,” “reliability,” and “neurogenesis.” These emerging trends vividly encapsulate the latest advancements in research and underscore the dynamic nature of investigations within this scientific landscape. Current hot topics include the HPA axis and probiotic therapies, indicating shifting research paradigms. As we delve deeper into the interplay between gut microbiota and depression, it becomes increasingly imperative to develop preventive and therapeutic strategies tailored to individual needs, paving the way for enhanced mental health outcomes.

Many pharmacological interventions for depression have proven to act via the gut microbiome, including both Western and traditional Chinese medicine. Evidence shows that commonly used antidepressants possess antimicrobial properties, particularly against Gram-positive bacteria (Ait Chait et al., 2020). Selective serotonin reuptake inhibitors (SSRIs), tricyclic antidepressants, and other drugs alter microbial diversity and composition. Specifically, SSRIs such as paroxetine and citalopram increase the abundance of *Eubacterium ramulus* in the gut, while tricyclic antidepressants elevate the levels of *Clostridium leptum* (Liu et al., 2023). Given that these bacteria produce the anti-inflammatory short-chain fatty acid butyrate during metabolism, their enhanced proliferation may offer supplementary benefits to depression treatments, thus improving drug efficacy. Furthermore, the gut microbiome can enzymatically modify the bioactivity and bioavailability of antidepressants, thereby influencing their effectiveness. For example, *Bacteroides thetaiotaomicron* and *E. coli IAI1* enhance the bioaccumulation of duloxetine, potentially diminishing its therapeutic efficacy by lowering its bioavailability (Klünemann et al., 2021). Traditional Chinese medicine has also found application in treating depression. Danshen’s bioactive compound, cryptotanshinone (CPT), reduces the harmful bacterial translocation associated with depression and exhibits promising antidepressant properties (Bian et al., 2023). The Zhi-zi-chi decoction (ZZCD), which includes Gardeniae Fructus (GF) and Semen Sojae Praeparatum (SSP), significantly influenced gut microbiota. Following ZZCD administration, dysbiosis caused by pathogenic bacteria linked to depression improved. In addition, the presence of beneficial probiotics increased at both the phylum and genus levels (Gao et al., 2023). Total glycosides from Cistanche stems can alleviate depression-like behaviors through bidirectional interactions between phytochemicals and the gut microbiota (Fan et al., 2021). Therefore, exploring the pharmacological effects of antidepressants from the perspective of the gut microbiome warrants further summarization, and bibliometric analyses in this area are also justified.

In summary, we analyzed the research trends regarding the correlation between gut microbiota and depression from literature over the past 5 years. This offers guidance for researchers in this field. Future studies could expand the database range and refine search strategies, thus providing more precise insights into the relationship between gut microbiota and depression.

## Data Availability

The original contributions presented in the study are included in the article/supplementary material, further inquiries can be directed to the corresponding author.
